# Community embedded reproductive health interventions for adolescents in Latin America: development and evaluation of a complex multi-centre intervention

**DOI:** 10.1186/1471-2458-13-31

**Published:** 2013-01-14

**Authors:** Peter Decat, Erica Nelson, Sarah De Meyer, Lina Jaruseviciene, Miguel Orozco, Zoyla Segura, Anna Gorter, Bernardo Vega, Kathya Cordova, Lea Maes, Marleen Temmerman, Els Leye, Olivier Degomme

**Affiliations:** 1 International Centre for Reproductive Health (ICRH), Ghent University, De Pintelaan 185 UZP 114, Gent 9000, Belgium; 2 Amsterdam University, Spui 21, Amsterdam, 1012WX, Netherlands; 3 Lithuanian University of Health Sciences (LUHS), Eiveniu str. 2, Kaunas, Lithuania; 4 Centro de Investigación y Estudios de la Salud (CIES), postal 3507, Managua, Nicaragua; 5 Instituto CentroAmericano de Salud (ICAS), postal 2234, Managua, Nicaragua; 6 University of Cuenca (UC), 12 of April, Cuenca, Ecuador; 7 South Group (SG), Calle Ecuador O-138, Cochabamba, Bolivia; 8 Department of Public Health, Ghent University, Watersportlaan 2, Gent, 9000, Belgium

**Keywords:** Reproductive health, Sexual behaviour, Adolescents, Latin America, Community based participatory research, Intervention mapping, Action research, Research design

## Abstract

**Background:**

Adolescents in Latin America are at high risk for unwanted and unplanned pregnancies, which often result in unsafe abortions or poor maternal health outcomes. Both young men and women in the region face an increased risk of sexually transmitted infections due to inadequate sexual and reproductive health information, services and counselling. To date, many adolescent health programmes have targeted a single determinant of sexual and reproductive health. However, recent evidence suggests that the complexity of sexual and reproductive health issues demands an equally multi-layered and comprehensive approach.

**Methods:**

This article describes the development, implementation and evaluation design of the community-embedded reproductive health care for adolescents (CERCA) study in three Latin American cities: Cochabamba (Bolivia), Cuenca (Ecuador) and Managua (Nicaragua). Project CERCA’s research methodology builds on existing methodological frameworks, namely: action research, community based participatory research and intervention-mapping.

The interventions in each country address distinct target groups (adolescents, parents, local authorities and health providers) and seek improvement of the following sexual health behaviours: communication about sexuality, sexual and reproductive health information-seeking, access to sexual and reproductive health care and safe sexual relationships.

In Managua, we implemented a randomised controlled study, and in Cochabamba and Cuenca we adopted a non-randomised controlled study to evaluate the effectiveness of Project CERCA interventions, in addition to a process evaluation.

**Discussion:**

This research will result in a methodological framework that will contribute to the improved design and implementation of future adolescent sexual and reproductive health interventions.

**Trial registration:**

ClinicalTrials.gov (NCT01722084)

## Background

In Latin America, 10 – 19 aged adolescents [1] are confronted with serious sexual and reproductive health (SRH) problems. Studies show that in Latin America adolescents initiate sexual activity at increasingly earlier ages and that only a minority of sexually active adolescents is taking any precaution for preventing sexually transmitted infections (STI) or pregnancy [2]. Data from 2001 showed that almost 50% of Nicaraguan women aged 20 – 24 gave birth for the first time before their 20th birthdate and a significant proportion of these pregnancies were unwanted [3]. Conception rates among sexually active single women aged 15–24 range from 14.1 per 100 woman-years in Nicaragua to 25.8 in Bolivia [2]. Early and unwanted pregnancies are linked to school drop-outs and have been steadily increasing among unmarried persons or those not in a stable union. A concurrent increase in abortion rates is to be expected. However, abortion is severely underreported because of its illegality in Latin American countries. In Brazil, Colombia, Dominican Republic and Peru between 10 and 21% of the hospitalisations for complications arising from unsafe abortion occurred among women aged 15–19 years [4]. These data suggest the number of induced abortions per 100 pregnancies ranges from 23 to 30 [4]. The magnitude of the STI and HIV epidemic in the region is also difficult to measure, due to limited data, underreporting, and weak surveillance systems [5].

Traditionally, adolescent health programmes aiming to prevent unwanted pregnancies and HIV/STI have targeted individual SRH issues despite the fact that changing health attitudes and behaviours demand a multidisciplinary and comprehensive approach [6]. For example, an intervention that proposes to improve access to SRH information and services may, in choosing such a unilateral approach, fail to achieve its desired objectives. Work in the field of HIV prevention has demonstrated that complex health issues which bisect socio-economic, geographic and gender inequities require culturally-informed, site-specific, and multidisciplinary responses [7]. Given the relatively new interest in adolescent sexual and reproductive health (ASRH) as a separate category within the broader field of maternal health and SRH, we do not yet have substantial evidence on what works best when taking a comprehensive approach [8]. The assessment of complex intervention strategies poses an additional challenge [9]. Studies which use an evaluation approach to measure impact of interventions in a continuously shifting social and cultural context are needed [10].

In response to this established need, we focus here on the development, implementation and evaluation design of the community-embedded reproductive health care for adolescents (CERCA) study (http://www.proyectocerca.org). Project CERCA is a multicentre study coordinated by the International Centre of Reproductive Health (ICRH) of the Ghent University. The CERCA study constitutes intervention research as it aims to develop and evaluate complex interventions that seek to improve access to, and the use of, SRH services by adolescents [11]. CERCA is based on the hypothesis that a comprehensive strategy of community-embedded interventions will improve the SRH and wellbeing of adolescents in target areas. The CERCA study, which runs from 2010 till 2014, will test this hypothesis in selected research settings in three Latin American cities: Cochabamba, Bolivia; Cuenca, Ecuador; and, Managua, Nicaragua. This intervention research will result in the development of a framework that will contribute to the planning of future SRH interventions which are both effective and responsive to target populations’ established needs.

## Methods

### Methodological frameworks

The CERCA study incorporates three methodological frameworks: action research, community based participatory research and intervention mapping.

**Figure 1:**
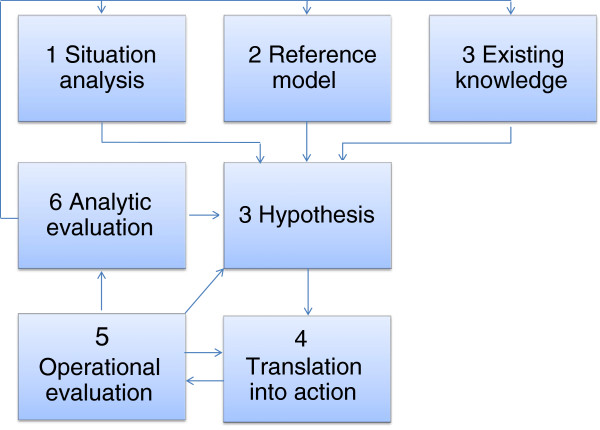
A basic methodological scheme for action research.

Action research (AR) provides the overarching framework. This methodology is most appropriate for intervention research projects as it enables the inclusion and testing of factors which are not necessarily quantifiable [12]. Nitayarumphong and Mercenier describe seven interacting stages in action research (see Figure 1). As the figure shows, these seven stages interact dynamically. For Project CERCA, we developed a hypothetical strategy for improving ASRH based on the following: 1) a study on ASRH determinants in each of the three study countries (stage 1 in the action research model); 2) a literature review of SRH and public health intervention models targeting adolescents both in Latin America and globally (stage 2); and finally, 3) a study of existing knowledge on adolescent SRH (stage 3). These studies formed the basis of empirical decision-making regarding the strategy (stage 4) and the design and implementation of intervention actions (stage 5). Stage 6 (evaluation of the implementation process) and Stage 7 (evaluation of project results) are forthcoming. 

In contrast to expert-driven health interventions, project CERCA seeks partnerships with local community residents in both the research and implementation phases of the project. Community-based participatory research (CBPR) is a collaborative research approach that is designed to ensure and establish structures for participation by all stakeholders in different aspects of the research process [13]. CBPR allows community members, health users and health providers to take ownership of the research and to critically reflect on iterative cycles of evaluation and monitoring. This approach enables a deeper understanding of the local context, as well as the creation of a more accurate framework for testing and adapting “best practices” to meet community’s needs. To this end community advisory boards have been established in all three Project CERCA sites. At the national level, staff from Ministries of Health, experts and members of international and local NGOs participate in the community board meetings, while at the local level community leaders, adolescents, youth educators, and parents are involved. These boards discuss monthly (local boards) or twice a year (national boards) the continuous process of understanding and of planning the intervention. The involvement of local health and government authorities in the CERCA advisory boards engenders their involvement in, and their support of, the project. Similarly, the ICRH project CERCA coordinators were guided in the project implementation and design by a local advisory board of Belgian experts (see acknowledgement).

When defining the intervention strategy (stage 4 of the action research process) it became clear to the CERCA consortium that the intervention-mapping (IM) tool would be key in the evidence-based development of CERCA interventions. Intervention mapping is a step-by-step process used in the development of health promotion programs [14]. These steps include: 1)a health needs assessment; 2) defining program objectives; 3) selecting appropriate theoretical models; 4) designing an intervention program; 5) adopting and implementing health intervention activities; and finally, 6) evaluation of health outcomes and intervention efficacy. There is an obvious overlap with the stages of Action Research discussed previously. For this study, we did not adhere to a single method but instead selected the Action Research framework to guide the research progress and the Intervention Mapping approach to guide the process of developing and selecting interventions as explained below.

This study is in compliance with the Helsinki Declaration on Ethical Principles for Medical Research Involving Human Subjects and is approved by the Bioethics Committee of Ghent University, Belgium (Belgian Registration number of the study: B670201111575).

### Development of intervention objectives

During the first year of Project CERCA we conducted a situation analysis in each of the study sites (Managua, Cochabamba and Cuenca) to assess the SRH needs and challenges of local adolescents. We collected both quantitative and qualitative data on SRH determinants, as well as relevant anthropological and sociological studies. The result was a comprehensive mapping of these health determinants which then helped identify specific health-seeking or health-impacting behaviours which could be addressed with Project CERCA interventions. Discussions in advisory boards, an advisory board and with CERCA consortium members led to the identification of a core set of project objectives: 1) adolescents communicate on their SRH with parents, partners and among peers; 2) adolescents access and receive accurate information on SRH; 3) adolescents make use of SRH services within primary health care; and 4) adolescents use consistently modern contraceptive methods.

Objective 1: Improved Communication on Sexual and Reproductive Health

Open and regular communication about SRH issues between adolescents and their parents has a protective effect [15]. Open communication at the family level can help to encourage adolescents to approach health care providers with questions and concerns related to their SRH [16] as well as encourage healthy sexual behaviour more generally [15,17,18]. Allen et al. found that adolescent girls who communicate easily with their mothers were considerably less likely to become pregnant [19] and Wilson et al. reported that open communication with parents led to postponed sexual debut and fewer unwanted pregnancies [20]. In semi-structured interviews conducted in the pre-intervention phase of Project CERCA, parents, young people, teachers and health providers in all three project sites acknowledged the difficulty and cultural taboos that prohibit the discussion of sex and sexuality within families, at school, and in the community as a whole. Similarly, other research has shown that in Nicaragua and Bolivia adolescents rarely, if ever, talk to their parents or other trusted adults (such as teachers) about sex or sexuality [21,22].

Objective 2: Improved Quality of, and Access to, Sexual and Reproductive Health Information

Interviewees in our study pointed out that sexual education at school is poor. Adolescents perceive what little SRH lessons they receive as negative, heavy on ‘scare tactics’ , moralistic and biologically oriented at the expense of discussions about relationships and communication. An independent assessment of sexual health education in Latin America and the Caribbean carried out by DeMaria et al. reports similar findings [23]. According to our interviewees the lack of comprehensive sexuality education and easy access to pornography and other dubious information sources lead to disinformation, poor knowledge on SRH, false beliefs, myths and negative attitudes towards e.g. contraception use. It is clear that young people need both accurate information on SRH, as well as the ability to navigate the overwhelming amount of inaccurate information in order to make healthy and well-informed choices [24].

Objective 3: Improved Access to Existing Sexual and Reproductive Health Services

Ideally, public health services are easily accessible and available to their target populations. However, it remains the case in a Latin American context that adolescents face multiple barriers when accessing public health care services [25]. Access in this region has been shown to be particularly problematic for young and unmarried women [26,27]. Studies also show that these barriers of access are not limited to the Latin American region specifically, but are in fact global in nature. The difficulties faced in accessing SRH services includes: 1) difficulty securing an appointment [25]; 2) concerns about confidentiality of care [28]; and, 3) concerns regarding communicating with health providers about SRH issues [29]. In addition, adolescents are negatively impacted by the following limitations of public health providers: 1) limited knowledge and training in the field of ASRH [25,30]; 2) lack of knowledge of legal provisions for confidential health services for adolescents [31,32]; and, 3) health provider reluctance to discuss SRH issues with adolescents [33]. The absence of a clear legal context creates an additional obstacle to improving adolescents’ access to SRH services in Bolivia and Nicaragua. A study in Nicaragua shows that adolescents who are able to make appointments with health providers are often not given the SRH care that they seek as health providers are unwilling to serve “legal minors” without the accompaniment of an adult guardian. Conversely, once these young women become pregnant their access to health services improves considerably regardless of their age as they can now be served under the auspices of the programma 'materno infantil' (mother and child care programme) [34].

Objective 4: Healthy and Safe Sexual Behaviours

A comparative analysis of data from Demographic and Health Surveys indicates that the percentage of sexually-active unmarried young women (ages 15 to 24) protected by contraception is 11.4% in Nicaragua and 19% in Bolivia [2]. A study among high school students in urban (Quito) and rural areas (Amazon Jungle) of Ecuador reported that 43% of the respondents had sexual intercourse of which 50% never used condoms and 70% did not use condoms at last intercourse [22,35].

There is substantial scientific evidence to support a multifaceted and community-centred approach when seeking to improve ASRH, both in terms of encouraging healthy behaviours [24] and improving access to existing SRH services [36]. On the one hand, public health interventions must achieve specific performance objectives related to attitudes, knowledge and practice in order to demonstrate the efficacy of a given approach. On the other, the nuances of local cultural norms and site-specific power dynamics related to socio-economic, racial and gender hierarchies must be taken into account for a given public health intervention to succeed. For example, in the preliminary phase of Project CERCA, ethnographic research consisting of one-to-one interviews, focus group sessions and participatory research methods revealed significant, though subtle, distinctions in local attitudes towards sexual diversity, abortion, acceptable female and male sexual behaviours, perceptions of existing health services and sex education. Ethnographic research conducted during the intervention period, to be published in the final year of the project, focuses more specifically on the impact of generation gaps on communication about sex and sexuality at the level of individual and extended families, and within the community more generally. As a result of the input from on-going ethnographic research in the form of quarterly focus group sessions with key community members (both young people and parents/grandparents of young people), the project has been able to tailor its communication and outreach campaigns to the unique needs of the communities in which we work, in addition to focusing on improving the sex and sexuality education of parents and significant adults.

In order to obtain a general overview of the global intervention plan, a different matrix has been developed for each of the three countries combining determinants, performance objectives and strategies for each intervention objective (Table 1, example of such a matrix).

**Table 1:** Matrix combining determinants, performance objectives and strategies

**Determinant**	**Performance objective**	**Strategy**
Awareness	Adolescents consider communication about SRH among peers as necessary and important.	Friends of youth (FoY) talk informally with adolescents about the importance of communicating about SRH
		Awareness raising during workshops and group discussions
		Awareness raising campaign on communicating about SRH (TV, radio, brochure, video presentation, movie showing, events and happenings in neighbourhoods, street theatre)
Introspection	Adolescents reflect upon their communication about SRH with peers.	Friends of youth (FoY) reflect individually with adolescents about communication behaviour
		Monitoring of own behaviour and social comparison during workshops and group discussions
		Testimonies
Skills	Adolescents have the skills to communicate with each other about SRH	Skills training in workshops
		Individual training by FoY
Social support	Social and cultural obstacles (existing myths, taboo, machismo and marianismo) are addressed.,	Support by peers during workshops and focus groups
		Individual support by FoY
		Activities in the communities and with parents. (see matrix communities and parents)

Example for the intervention objective 'promoting communication on sexual and reproductive health among peers'.

### Theory-informed intervention methods and practical strategies

In order to achieve these stated project objectives, we identified relevant theoretical models and strategies. Specifically, we selected the Theory of Planned Behaviour (TPB) and the Social Cognitive Theory (SCT) as frameworks for the development and design of intervention strategies. Recent studies have demonstrated the ways in which both theories can be used to successfully promote safe sexual behaviour [37]. For example, the TPB^a^ has been proven an effective model for influencing adolescents’ behaviour with regards to modern contraceptive method use and health seeking behaviours. The SCT helped us in the development of strategies at interpersonal level namely to improve communication about sex and adolescent sexuality at the level of families, communities, and within public health services. In addition, we conducted discussion groups with key stakeholders in the three project sites to brainstorm on possible interventions. Using the suggestions for interventions from the stakeholders, the mentioned theoretical frameworks and the SRH determinants we identified in Phase 1 of the project, we then drew up an intervention plan of action. This dialogical process has continued to play a dynamic role in the continuous adaptation and modification of intervention strategies, in combination with the results from monitoring and evaluation activities and input from community-based ethnographic research.

### Intervention strategies targeting adolescents

In Managua, due to the relatively high incidence of young people out-of-school in the selected low-income target neighbourhoods, the local partner chose to carry out intervention activities at the community level (e.g. mobile cinemas, sporting events, door-to-door outreach and education campaigns). In Cuenca and Cochabamba, the consortium partners chose a high school-focused intervention strategy, conducting SRH workshops and facilitating youth groups in classrooms and school auditoriums. In Managua Friends of Youth (FoY)^b^ were selected. FOY are young adults, intensively trained in SRH [38]. They served as mentors of the adolescents in their community, helped them building their competence to make deliberate choices and when needed they referred and eventually accompanied adolescents to an appropriate health provider. Besides the one-to-one interaction with adolescents, the FoY also supported community activities including workshops, exhibitions, street theatre, movie showing and awareness campaigns. The FoY were supervised by the programme implementers of the research team. They received small financial incentives. In Cuenca and Cochabamba there was not a FoY strategy in place. Young professionals, psychologists and social workers, organized similar activities as the FoY did in Managua. In Cuenca young people participated in capacity building exercises so that they might provide peer support on SRH issues with their friends and schoolmates. New media were also extensively used in the intervention strategy to reach adolescents, particularly Facebook and cell-phone messages. Those communication methods were used to a lesser extent in Managua compared to Cuenca and Cochabamba, as adolescents from low income neighbourhoods in Nicaragua have less access to those new media.

### Intervention strategies targeting parents and adult family members

In each of the three project sites, consortium partners brought the message of Project CERCA to the parents, grandparents and significant adults of adolescents through a combination of media campaigns, workshops (at schools, health centres and community centres,) and discussion groups. In Managua, ICAS creatively negotiated the initial resistance to open discussion of ASRH issues by carrying out home visits and informal talks in target neighbourhoods. Similarly, in Cuenca, the university of Cuenca recruited parents following blanket workshops at local high schools to participate in discussion groups and share information on the project with friends and neighbours.

### Intervention strategies targeting health providers and health centres

Interventions included workshops and virtual learning activities. Health providers were trained in patient centeredness focusing on characteristics of a good provider-patient communication such as empathy, courtesy, friendliness, reassurance, support, encouraging patients’ participation, giving explanations, positive reinforcement, shared decision making and patient-centred verbal styles. An educational program with role plays, videotaped vignettes of simulated patients and feedback on own recorded consultations has been implemented to teach communication skills. In order to improve other competences among providers, existing national and international guidelines on providing sexual health care services to adolescents were presented and discussed during workshops. In peer sessions they were encouraged to reflect on own attitudes, values and beliefs on adolescent sexuality and how these may affect their work with adolescents. In case of casual problems with the supply chain Project Cerca ensured the permanent availability of contraceptives in the health centres. Concurrently, outreach activities were intensified. Health providers paid visits to schools and communities and offered counselling and family planning methods to adolescents.

### Intervention strategies targeting local authorities

In order to better involve local authorities in Project CERCA (public health officials, religious leaders, school prefects, municipal and regional government representatives) consortium partners have conducted continuous information and outreach campaigns. Specifically, they have carried out both formal and informal visits to discuss CERCA objectives, organized information events, produced and disseminated newsletters and reports, and in Cuenca and Cochabamba, helped to form an SRH advocacy and advisory committee. These actions endeavoured to increase knowledge of Project CERCA objectives and strategies and to encourage pro-adolescent SRH decision-making and policy changes at the local, municipal and regional level. The CERCA consortium believes that through outreach activities and on-going dialogue with local decision-makers we will help impact on adolescent SRH generally through increased awareness of the key issues and the reduced resistance to ASRH education and services.

### Intervention strategies targeting community members

CERCA consortium partners in all three cities have sought to create an environment conducive to health adolescent sexual and reproductive behaviours by encouraging positive changes in attitudes, knowledge and practice at the community level. Specific intervention strategies include the organization of sports and cultural events where CERCA information is distributed and promoted; the implementation of SRH education and awareness media campaigns and community health fairs, and by continuously seeking the involvement of local community members in CERCA activities. In Managua, ICAS sponsored a soccer tournament involving competing target neighbourhoods, garnering significant local media coverage in the process. In Cuenca, University of Cuenca sponsored events such as a 10k running race and a youth SRH video competition to build awareness and support for the project. In Cochabamba, the South Group collaborated with other local SRH-focused NGOs to host an HIV/AIDS and SRH awareness fair for all area high schools. In addition to these on-the-ground activities, Project CERCA partners have used Facebook, text messaging and radio campaigns to keep community members up-to-date on project activities.

In summary, the Project CERCA intervention has incorporated the following strategic elements in an effort to improve ASRH in select communities:

1) PARTICIPATORY APPROACH:

The core principle of Project CERCA is “community-embeddedness”, which means developing and implementing project objectives in close collaboration with adolescents, parents/grandparents and family members of adolescents, health providers, teachers, local leaders and public health authorities in each of the selected project sites. In practice, the degree of local stakeholder participation has varied due to the different degrees of previous participatory-approach experience among consortium partners. However, all consortium partners have endeavoured to develop and implement project activities with the support and input of community members with the aim of increasing the efficacy and sustainability of project interventions.

2) HARMONISATION WITH EXISITING HEALTH SYSTEMS AND GOVERNMENT POLICIES:

In addition to seeking the collaboration of key stakeholders through participatory project design and implementation processes, Project CERCA has also sought to develop intervention activities in line with existing health system structures and government policies. Given the diverse nature of health systems and policies in each of the three CERCA countries, the strategies adopted look quite different. In Managua, for example, the public health system provides the majority of health services and private health services are either unaffordable or unavailable in the neighbourhoods selected for participation. Consequently, the project could identify which health centres adolescents living in target areas would access for SRH services. However, the recently implemented Family and Community Health Model in Nicaragua has meant that CERCA partner CIES could not set up an adolescents-only clinic within these identified health centres, providing instead capacity-building in ASRH for public health providers [39]. In Cuenca and Cochabamba, however, private health services exist in parallel with the public health system and are commonly used by adolescents. This has resulted in generalized communication and outreach campaigns on ASRH issues and modern contraceptive use in order to reach those adolescents not using the public health system for their SRH needs. Lastly, CERCA consortium partners have sought collaboration with ministry of health officials in all three countries, sharing research results and reports in an effort to promote ASRH-friendly policies.

3) FOCUS ON GENDER:

Gender was a transversal topic throughout the intervention process as there is evidence that the more gender considerations are integrated and explicitly addressed within programmes, the greater is the likelihood of improved SRH outcomes for both young men and women [40]. In practice considering the gender topic within the project referred mainly to the improvement of the equality between boys and girls and taking into account the different perspectives and experiences of both girls and boys, women and men. Between countries, there were methodological and content differences in treating the gender topic. This is a logical consequence of the different perspectives and experiences that exist among and within local teams which reflects the societal diversity. During consortium meetings gender aspects and other ethical issues were repeatedly discussed. This resulted in a progressive evolution towards more converging attitudes and viewpoints.

4) MULTIDISCIPLINARY PROJECT TEAMS:

Project CERCA has drawn on multiple disciplines, including western medicine, epidemiology, sociology, anthropology, demography and political sciences, in the design and development of each city-specific intervention strategy. While the content and the focus of each intervention strategy varied according to the disciplinary strengths of the local teams, all members of the CERCA consortium have worked to incorporate multiple perspectives on ASRH in their decision-making processes. This has required team members to stretch outside of disciplinary comfort zones and think in new ways about the challenges of ASRH specific to their target areas.

### Implementation and monitoring

In all Project CERCA intervention sites, consortium members created trimester and annual operational plans in collaboration with key stakeholders. These plans were based on the performance objectives and determinant factors matrices. The involvement of community members in the planning process helped to identify potential barriers to change and possible ways of managing these challenges. We also developed a monitoring and evaluation protocol to provide additional input to the action-research process, enabling CERCA partners to identify any changes needed to make intervention activities work. This M&E data includes information on target groups, duration of activities, activity objectives and determinants, methodologies used, activity content, number of participations, as well as general observations and comments. Each trimester the intervention components are then collated into a summary activity form, which allows the CERCA consortium to identify any gaps in the overarching strategy. In addition, the primary health centres involved in Project CERCA agreed to ASRH service-registration protocols. Finally, ethnographic fieldwork consisting of in-depth interviews (both video and audio), participant observation, community discussion groups, and participatory research methods, has engendered a more nuanced understanding among consortium members of the cultural contexts in which the intervention activities are taking place. This research has also encouraged greater dialogue between the target populations of the project (adolescents, families of adolescents, and health providers) and the consortium members responsible for intervention decision-making.

### Evaluation

In order to measure the impact of Project CERCA interventions, consortium partners have carried out a pre-intervention baseline survey of both target groups and control groups, and will conduct a post-intervention survey at the 18-month mark.

In Managua, we applied a cluster randomized controlled study, with results to be published according to the CONSORT statement [41]. For this study we used the town district boundaries as our selection unit, calculating sample size based on the outcome of modern contraceptive use. Drawing from national demographic statistics [42], we estimated that from a current 13–18 age cohort of adolescents in Managua 50% will be sexually active after two years and that 30% of the sexually active adolescents use a modern contraceptive with a 0.01 intra-cluster correlation within town districts. We assumed an average cluster size of 170 adolescents participating in the surveys. To detect a significant difference in contraceptive use of 10% between the intervention and control groups, we determined that 18 clusters (6 intervention and 12 control) were necessary. A list with population data of all the town districts in Managua, based on a census of 2005, was obtained from the municipality. We took into consideration only those 33 town districts which comply with the previously defined inclusion criteria (between 1400 and 4500 inhabitants and with more than 50% poor people –poverty measurement through the Unsatisfied Basic Needs approach-). A multilevel sampling method was applied to select the intervention and control group from these districts. Firstly, two intervention public primary health centres were randomly chosen from the nine public primary health centres in Managua. Subsequently, among the twelve town districts served by those two health centres, six centres were randomly selected. From the 21 remaining town districts served by other than the intervention health centres twelve were randomly selected as control groups. Trained interviewers conducted surveys among adolescents aged 13 to 18 in control and intervention districts by going door-to-door. After obtaining verbal consent from the adolescents and the responsible adult, when present, the interviewer began the questionnaire. For those questions directly related to sexual behaviour, adolescents completed the questionnaire by self-administration. These same adolescents will be interviewed a second time at the 18-month intervention mark.

In Cuenca and Cochabamba, consortium partners carried out the survey at local high schools. Random allocation to an intervention and control group was not possible as the intervention could only be embedded in health centres that were allied with the research group. Within the intervention area, the university of Cuenca team chose to carry out intervention activities at three high schools, and the South Group team in Cochabamba chose to work with seven high schools. For each intervention school the local research team enrolled a control school with similar characteristics located in a separate district. All parents or guardians of the students from the selected schools were informed about the survey and were given the possibility to refuse the enrolment of their child or protected minor in the study. At selected schools interviewers visited all classrooms and invited all students aged 13 to 18 (whose parents did not oppose) to fill out the self-administered questionnaire after signing an informed consent. The same procedure will be repeated at the 18 intervention month-mark.

The questionnaire was designed by CERCA consortium members taking into account the intervention objectives and the determinants of adolescents’ SRH identified during the situation analysis. It was based on the illustrative questionnaire for interview-surveys with young people conceived by John Cleland for the Word Health Organization. The questionnaire contained questions regarding intervention goals, SRH history, self-esteem, gender norms, and social determinants of SRH behavior. The main outcome indicators were adapted to the specific cultural, language, and demographic context and constructed to assess the impact of the CERCA intervention on adolescent’s behaviour with regards to the following: 1) access to accurate information on SRH; 2) degree of comfort and extent of communication on issues of sex and sexuality; 3) use of existing SRH services; and finally, 4) use of modern contraceptive methods. For the determinant variables related to gender attitudes and self esteem we used, respectively, the Spanish version of the validated “attitude towards women scale for adolescents” [43] and the questions on self-esteem from the JOP monitor 2 questionnaire [44]. The questionnaire was piloted in each country by presenting it to 30 adolescents not belonging to the sample population and then asking their opinion of the content, clarity and length of the questionnaire. Following the second round of surveying at the 18-month mark, we will use multilevel regression models to evaluate the impact of the CERCA intervention, adjusting for cluster effects within districts (Managua) and in schools (Cochabamba and Cuenca). Analyses will be performed according to the intention-to-treat principle. Linear and regression analyses will assess the variation of outcome values over time in intervention and control groups.

## Discussion

Project CERCA aims to develop, implement and test an intervention model capable of improving ASRH in three Latin American cities. We designed the research framework using the existing methodologies of action-research, community based participatory research and intervention mapping. These methodological tools allow us to conduct, on the one hand, intervention research that accounts for the complexity of ASRH determinants; and on the other, to carry out a comprehensive intervention strategy and evaluation plan. Instead of hewing precisely to one specific methodological framework, the CERCA consortium opted develop a research and intervention model drawing from all three (AR, CBPR, and Intervention Mapping) in order to best meet existing ASRH needs, to ensure community ownership and participant empowerment, and to be responsive to changing political and socio-cultural contexts. In order to determine the effectiveness of the interventions it was crucial to work out an evaluation approach that allows us to measure impact of interventions in a continuously changing context [45]. To that end we developed a specific controlled impact study in the three cities making use of contextualized measurable behaviour outcomes. Given the complexity and variability of interventions, we expect to evaluate the effectiveness of the intervention model as a whole rather than attributing results to a single intervention activity or strategy. Whereas the quantitative evaluation purports to demonstrate the potential efficacy and impact of the intervention strategy, ethnographic (qualitative) research will provide a deeper understanding of the complex and community-specific factors that have challenged the intervention process. The monitoring data will be used for the detailed description of the intervention process, information which will be essential for reproducing the strategy in other contexts or on a larger scale.

The results of the CERCA study will help to gain insight in how to design effective health interventions targeted to ASRH needs specifically, and more generally will help to better understand how existing public health systems can be more responsive to the changing needs and demands of an adolescent population. Complete results from Project CERCA will be available in 2014.

## Endnotes

^a^ According to the TPB, individuals are able to make a deliberate choice to behave in a certain way. This choice depends on attitudes towards the behaviour, social influences (social norms, reinforcement, tradition and culture), the perceived belief in oneself to make a deliberate choice and external factors. SCT focuses on observational learning and self-determination, namely the interactions of the person and the environment, expectations and self-efficacy.

^b^ The term FoY (Friends of Youth) was taken from a successful project implemented by Population Council in Kenya. FoY are young people slightly older and more experienced in relation to SRH who provide information to adolescents, promote healthy sexual behaviour, serve as a mentor and person to turn to when adolescents have SRH questions as well as to refer and eventually accompany adolescents to an appropriate health provider.

## Competing interests

The authors declare that they have no competing interests.

## Authors' contributions

The work presented here was carried out in collaboration between all authors. PD, EN, SD, LJ, MO, ZS, AG, BV, KC, LM, EL, MT and OD provided support in the design of the study and contributed intellectual input into the main ideas of this paper. PD, SD and LJ designed and coordinated the implementation of the intervention. PD, MO, ZS, BV and KC supervised the data-collections and the implementation of the intervention. PD drafted the manuscript. EN provided substantial content and rewriting support. All authors contributed to the drafting of the manuscript. PD will act as guarantor of the paper. All authors read and approved the final manuscript.

## Pre-publication history

The pre-publication history for this paper can be accessed here:

http://www.biomedcentral.com/1471-2458/13/31/prepub
